# Characterization of High-Grade Glial and Related Neuroepithelial Tumors Using the AOSNP-ADAPTR Framework: A Case Series With Review of Literature

**DOI:** 10.7759/cureus.113142

**Published:** 2026-07-22

**Authors:** Soumyanil Pandey, Alka Dixit Vats, Adreena Mittal, Swati Singh

**Affiliations:** 1 Pathology, Santosh University, Ghaziabad, IND

**Keywords:** aosnp-adaptr, diffuse astrocytic tumor, gfap, glioblastoma, high-grade glioma, idh1, immunohistochemistry, ki-67, olig2, who cns5

## Abstract

High-grade glial tumors are among the most aggressive primary neoplasms of the central nervous system and are associated with substantial morbidity, rapid progression, and poor survival. The classification of high-grade glial tumors requires a diagnostic approach combining histomorphology with immunohistochemical and molecular findings. In resource-constrained settings, the AOSNP-ADAPTR (Asian Oceanian Society of Neuropathology - Adapting Diagnostic Approaches for Practical Taxonomy in Resource-Restrained Regions) framework provides a practical strategy for implementing these principles using available diagnostic tools. This case series included five adult patients with intracranial tumors showing high-grade glial or related neuroepithelial morphology on histopathological examination. Clinical presentation, radiological findings, gross features, microscopic morphology, and available immunohistochemical findings were evaluated. Markers used or recommended included GFAP, Olig2, S-100, IDH1 R132H, p53, ATRX, Ki-67, EMA, D2-40, CD56, and cytokeratin markers, wherever indicated. Some immunohistochemistry (IHC) images included in this manuscript were obtained from an external referral laboratory because the corresponding IHC markers were not available at our institute. Consequently, these images contain the original laboratory watermark and have been retained in their original form to preserve image authenticity. All five patients were male, with ages ranging from 45 to 84 years. The common presenting features included headache, seizures, focal neurological deficits, lethargy, and symptoms of raised intracranial pressure. Four cases showed histomorphological features consistent with a diffuse high-grade glial neoplasm with necrosis and microvascular proliferation. Among these, three cases were in keeping with glioblastoma-like morphology, while one IDH1 R132H-positive tumor required integrated interpretation under the fifth edition of the WHO Classification of Central Nervous System Tumors (WHO CNS5) terminology and was appropriately classified as astrocytoma, IDH-mutant, CNS WHO grade 4 rather than glioblastoma. One additional case showed perivascular pseudorosettes and ependymal canal-like structures, raising the possibility of a high-grade ependymal or glial neoplasm and warranting further immunohistochemical categorization. The cases illustrated the diagnostic utility of combining histopathology with a targeted IHC panel in a resource-adapted setting. This case series demonstrates the clinicopathological and immunohistochemical spectrum of high-grade glial and related neuroepithelial tumors. IDH1 R132H IHC was critical in reclassifying one morphologically grade 4 tumor as an astrocytoma, IDH-mutant rather than a glioblastoma. Aberrant CK7 expression in one case highlighted the need for expanded IHC to exclude metastatic carcinoma. The AOSNP-ADAPTR framework enables WHO CNS5-aligned classification using targeted IHC panels in resource-constrained settings.

## Introduction

Gliomas constitute the most common primary malignant brain tumors in adults, with glioblastoma (GBM) being the most frequent and aggressive subtype. High-grade glial tumors are histologically characterized by hypercellularity, nuclear pleomorphism, brisk mitotic activity, microvascular proliferation, and pseudopalisading necrosis [1,2]. Significant morphological overlap with metastatic carcinoma and other high-grade malignancies necessitates immunohistochemical and molecular characterization for accurate diagnosis and classification [3].

The fifth edition of the WHO Classification of Central Nervous System Tumors (WHO CNS5, 2021) mandates an integrated diagnostic approach incorporating histomorphology with molecular parameters including IDH mutation status, ATRX expression, 1p/19q co-deletion, and CDKN2A/B homozygous deletion [4,5]. IDH-wildtype diffuse astrocytic tumors fulfilling grade 4 histological criteria are now classified as "glioblastoma, IDH-wildtype," whereas IDH-mutant tumors with identical histological features carry the distinct designation of "astrocytoma, IDH-mutant, CNS WHO grade 4" - a distinction with significant prognostic and therapeutic implications [6]. Cases with unavailable or discordant molecular data are accommodated by the NOS (not otherwise specified) and NEC (not elsewhere classifiable) suffixes [7].

Immunohistochemical markers, including GFAP, Olig2, S-100, IDH1 R132H, p53, ATRX, and Ki-67, are indispensable for confirming glial lineage, assessing proliferative index, and serving as surrogate molecular indicators when comprehensive molecular profiling is unavailable [8,9]. In resource-constrained settings, the AOSNP-ADAPTR (Asian Oceanian Society of Neuropathology - Adapting Diagnostic Approaches for Practical Taxonomy in Resource-Restrained Regions) framework provides a structured approach to implementing WHO CNS5 principles using targeted immunohistochemistry (IHC) panels, enabling clinically meaningful classification without full molecular testing [10].

The present case series describes five adult males with high-grade glial and related neuroepithelial tumors evaluated at a tertiary care center, highlighting their clinicopathological features, IHC findings, and integrated WHO CNS5 classification within the AOSNP-ADAPTR framework.

## Case presentation

Case 1

A 75-year-old male presented with clinical features suggestive of a right frontal lobe space-occupying lesion. His symptoms included headache, focal neurological deficits, and features of increased intracranial pressure. Radiological evaluation revealed a mass lesion in the right frontal region, following which craniotomy and tumor decompression were performed.

**Figure 1:**
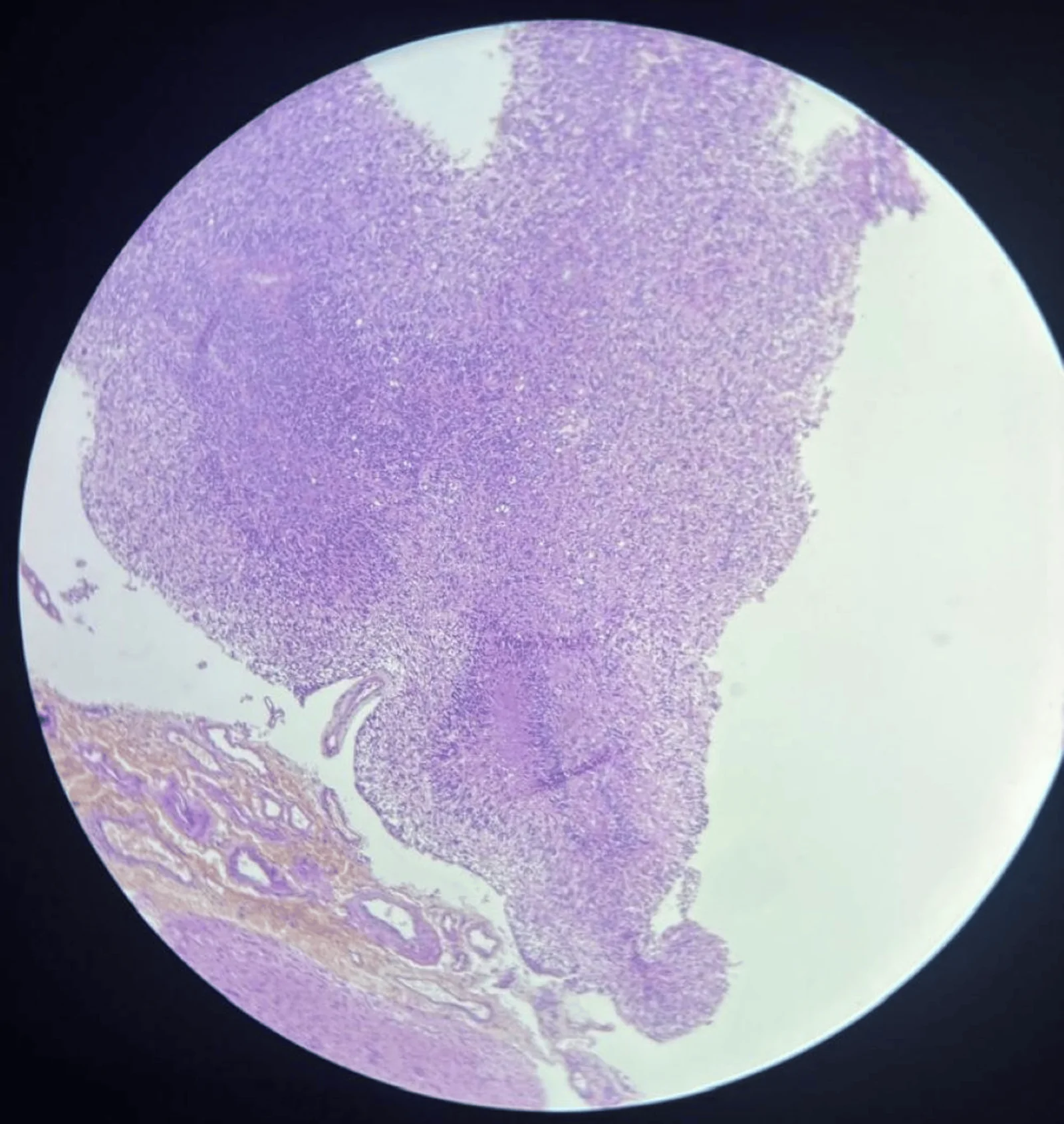
Low-power photomicrograph showing a hypercellular infiltrative glial neoplasm involving the brain parenchyma with diffuse growth, consistent with a high-grade diffuse astrocytic neoplasm (H&E, 10×).

**Figure 2:**
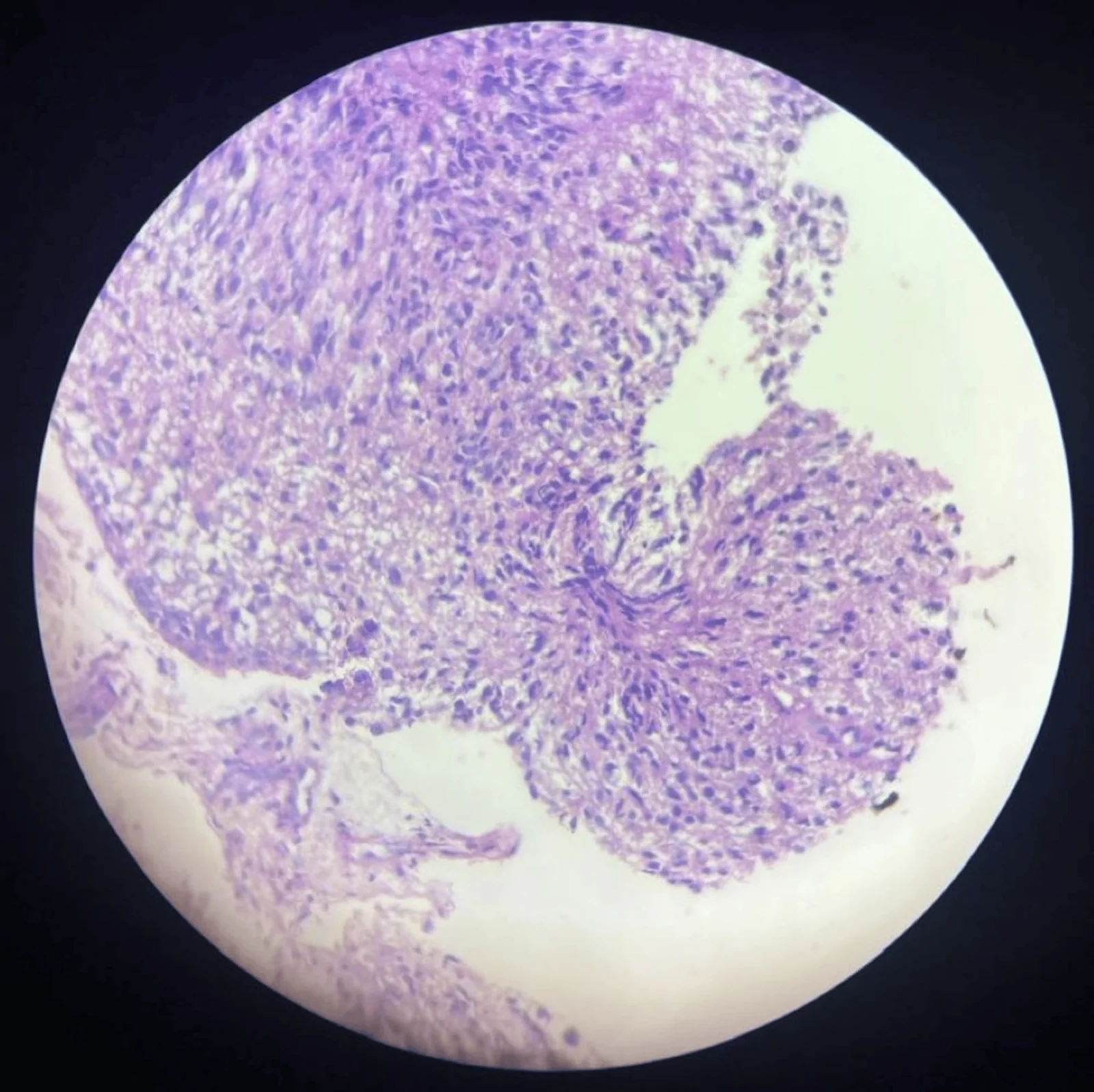
Photomicrograph showing a hypercellular infiltrative glial neoplasm composed of pleomorphic tumor cells with hyperchromatic nuclei and increased cellularity, consistent with a high-grade diffuse neoplasm (H&E, 40×).

On gross examination, multiple grayish-brown tissue fragments measuring 5 × 2 × 1 cm in aggregate were received. Histopathological examination showed a highly cellular infiltrating neoplasm involving the brain parenchyma (Figures 1, 2). The tumor cells displayed moderate-to-marked pleomorphism and increased mitotic activity, with approximately 3-4 mitoses per 10 high-power fields. Areas of necrosis with a pseudopalisading arrangement of tumor cells were noted. Additional findings included focal hyalinization and hemorrhagic areas.

IHC showed positivity for GFAP, IDH1 R132H, p53, and Ki-67. These features support the diagnosis of a high-grade diffuse astrocytic neoplasm with IDH1 R132H positivity. In view of the WHO CNS5 terminology, such a lesion should not be automatically labeled as GBM if an IDH mutation is confirmed. If an integrated diagnostic evaluation confirms an IDH-mutant diffuse astrocytic tumor with grade 4 histological features, the appropriate category would be astrocytoma, IDH-mutant, CNS WHO grade 4.

Further correlation with ATRX status and, where feasible, additional molecular evaluation is recommended for complete integrated classification.

Case 2

A 52-year-old male presented with a six-month history of seizures, lethargy, and headache. Neuroimaging demonstrated a left fronto-parietal lesion, with possible extension toward the right temporal lobe, raising radiological suspicion of a high-grade glioma. He underwent craniotomy with tumor decompression, and tissue from the left parietal region was submitted for histopathological evaluation.

**Figure 3:**
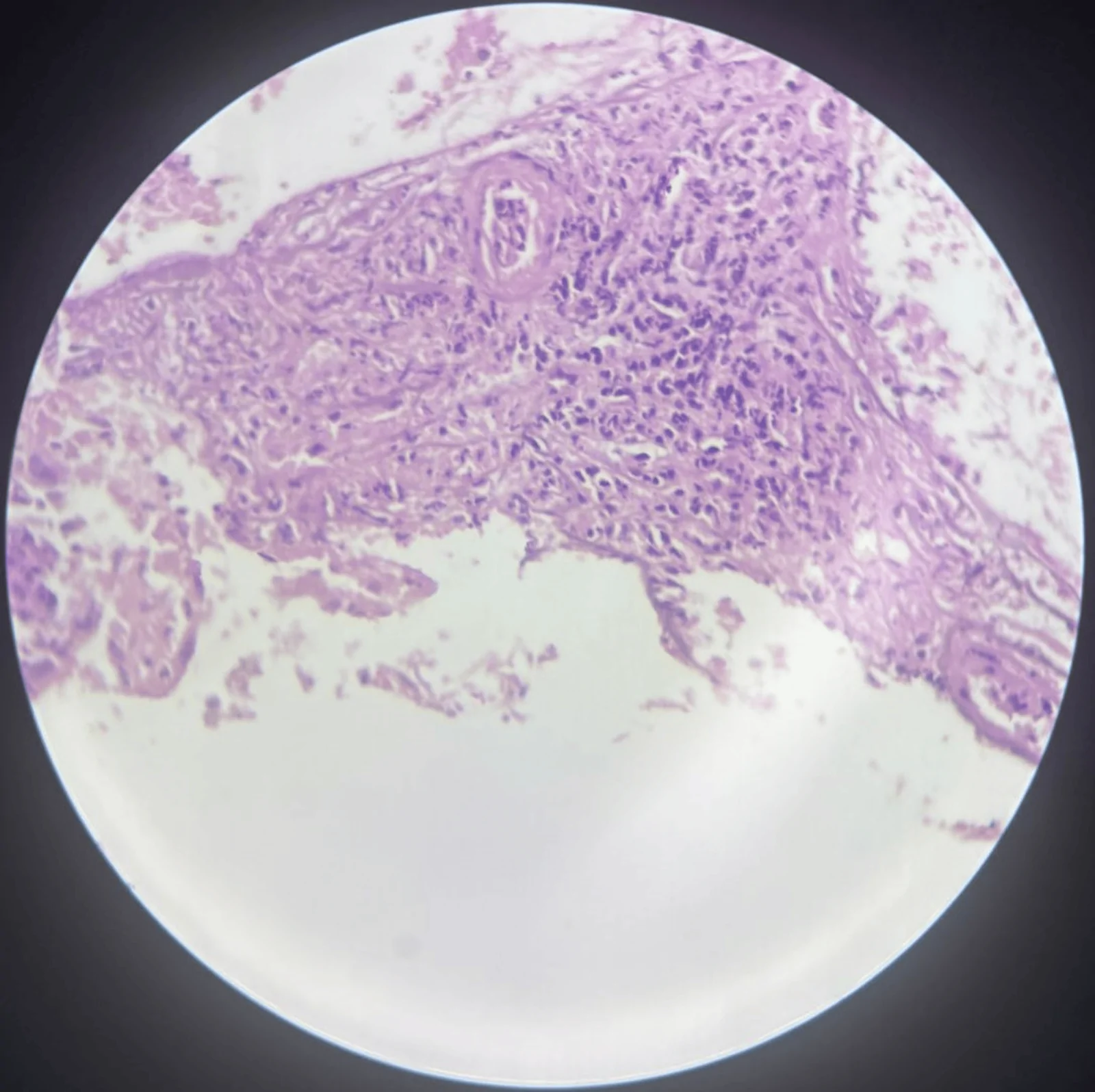
Photomicrograph showing prominent microvascular proliferation with thick-walled hyalinized blood vessels embedded within a hypercellular glial neoplasm, a characteristic feature of high-grade glioma/glioblastoma (H&E, 40×).

Grossly, the specimen consisted of multiple grayish-brown soft tissue fragments measuring 4 × 3 × 1 cm. Microscopic examination revealed a highly cellular malignant neoplasm composed of markedly pleomorphic tumor cells with overlapping nuclei, clumped chromatin, and conspicuous nucleoli. Extensive vascular proliferation with dilated and congested blood vessels was noted. Prominent microvascular proliferation with thick-walled hyalinized blood vessels was also identified (Figure 3). Multiple areas of tumor necrosis with a pseudopalisading arrangement were present. Hemorrhage and mixed inflammatory infiltrates were also identified.

Based on these features, a diagnosis of high-grade diffuse glial neoplasm with morphological features favoring GBM was considered. IHC showed GFAP positivity and IDH1 R132H negativity (IDH-wildtype), supporting the classification of GBM, IDH-wildtype, CNS WHO grade 4 in the appropriate integrated diagnostic context.

Case 3

An 84-year-old male presented with a left frontoparietal space-occupying lesion clinically suspected to represent a glioma. Tumor excision was performed, and tissue was submitted for histopathological examination.

Grossly, a single yellowish-brown soft tissue specimen measuring 2.8 × 2.6 × 2.5 cm was received, showing solid yellowish-white areas. Microscopic examination revealed a highly aggressive malignant neoplasm composed of tumor cells arranged in sheets, clusters, and scattered singly. The tumor cells demonstrated marked pleomorphism, enlarged nuclei, vesicular chromatin, prominent nucleoli, and moderate granular eosinophilic cytoplasm. Multinucleated giant cells were identified. Extensive necrosis, microvascular proliferation, and brisk mitotic activity, with approximately 5-7 mitoses per 10 high-power fields, were present.

The morphology raised a differential diagnosis of a high-grade diffuse glial neoplasm/GBM-like tumor and metastatic malignant neoplasm.

**Figure 4:**
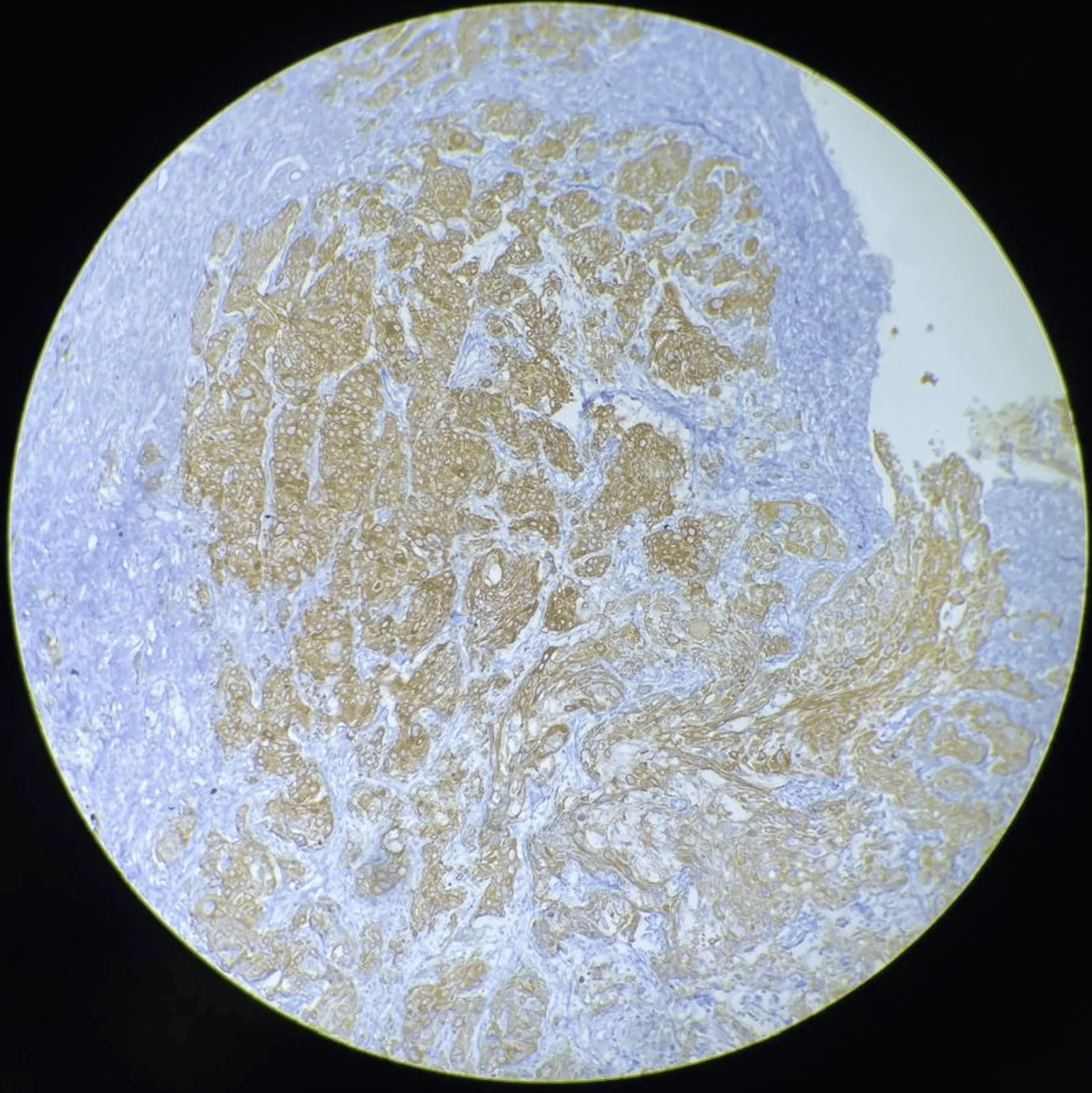
Pan-cytokeratin (Pan-CK) immunohistochemistry showing cytoplasmic positivity in tumor cells.

IHC showed GFAP positivity and Olig2 positivity, supporting glial differentiation. IDH1 R132H IHC was negative, suggesting IDH-wildtype status. Pan-cytokeratin IHC showed cytoplasmic positivity in tumor cells (Figure 4). However, CK positivity should not be interpreted as direct support for GBM; rather, it warrants cautious evaluation in conjunction with the complete immunophenotypic profile, radiological findings, and clinical correlation to exclude metastatic epithelial malignancy or aberrant cytokeratin expression.

**Figure 5:**
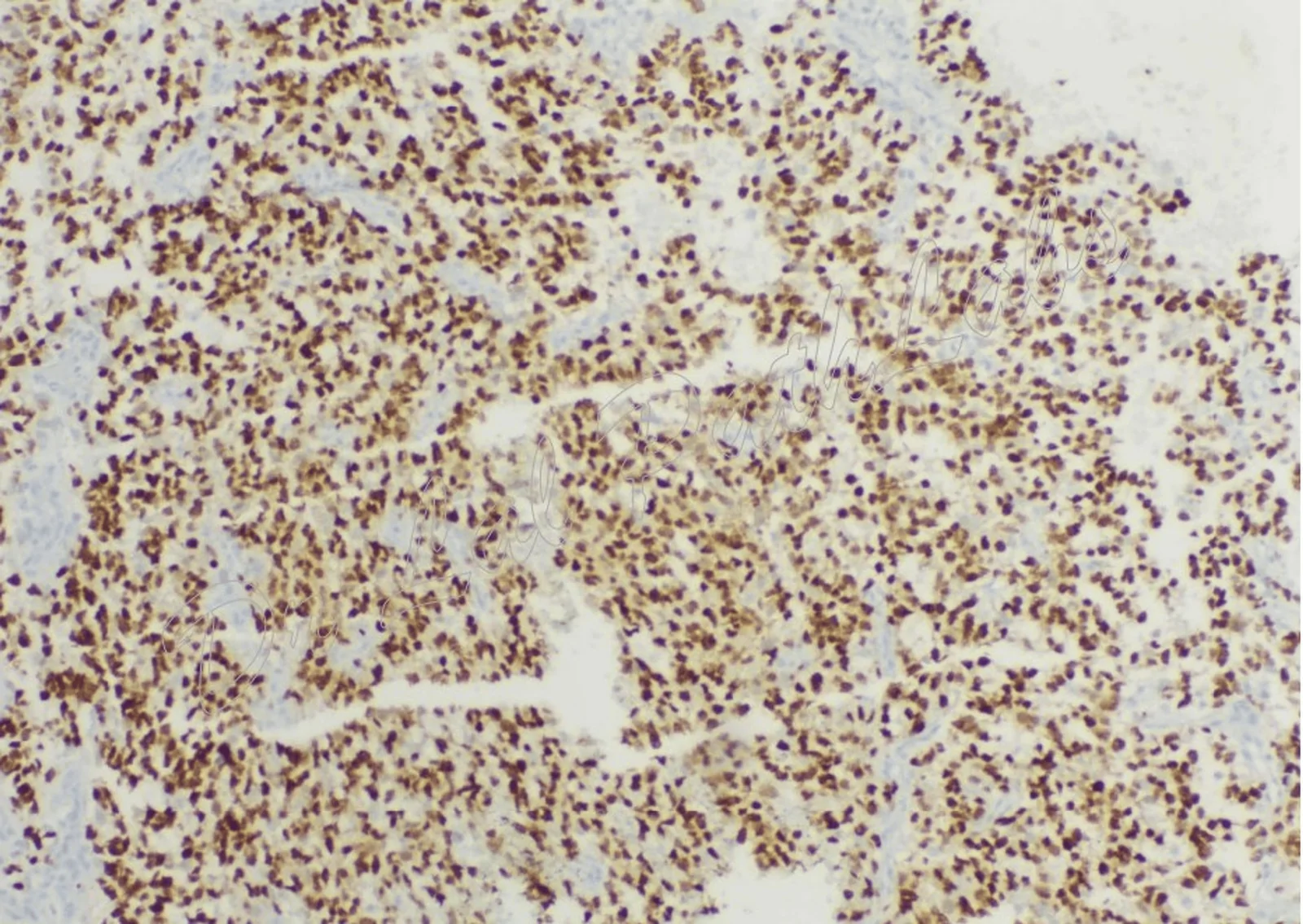
OLIG2 immunohistochemistry demonstrating diffuse and strong nuclear positivity in neoplastic cells, supporting glial lineage differentiation in a high-grade diffuse glial neoplasm with glioblastoma-like morphology (IHC, ×100). The image has been outsourced.

Olig2 IHC demonstrated diffuse and strong nuclear positivity in neoplastic cells, further supporting glial lineage differentiation in this high-grade diffuse glial neoplasm with GBM-like morphology (Figure 5).

A more appropriate final wording would be "high-grade malignant neoplasm with glial differentiation, favoring GBM-like diffuse glial tumor; metastatic carcinoma to be excluded by clinicoradiological correlation and expanded immunohistochemical work-up where indicated."

Case 4

A 45-year-old male with a history of generalized tonic-clonic seizures and neurocysticercosis presented with a right fronto-temporal space-occupying lesion. Radiological evaluation suggested a GBM with a satellite lesion. Craniotomy with tumor decompression was performed.

**Figure 6:**
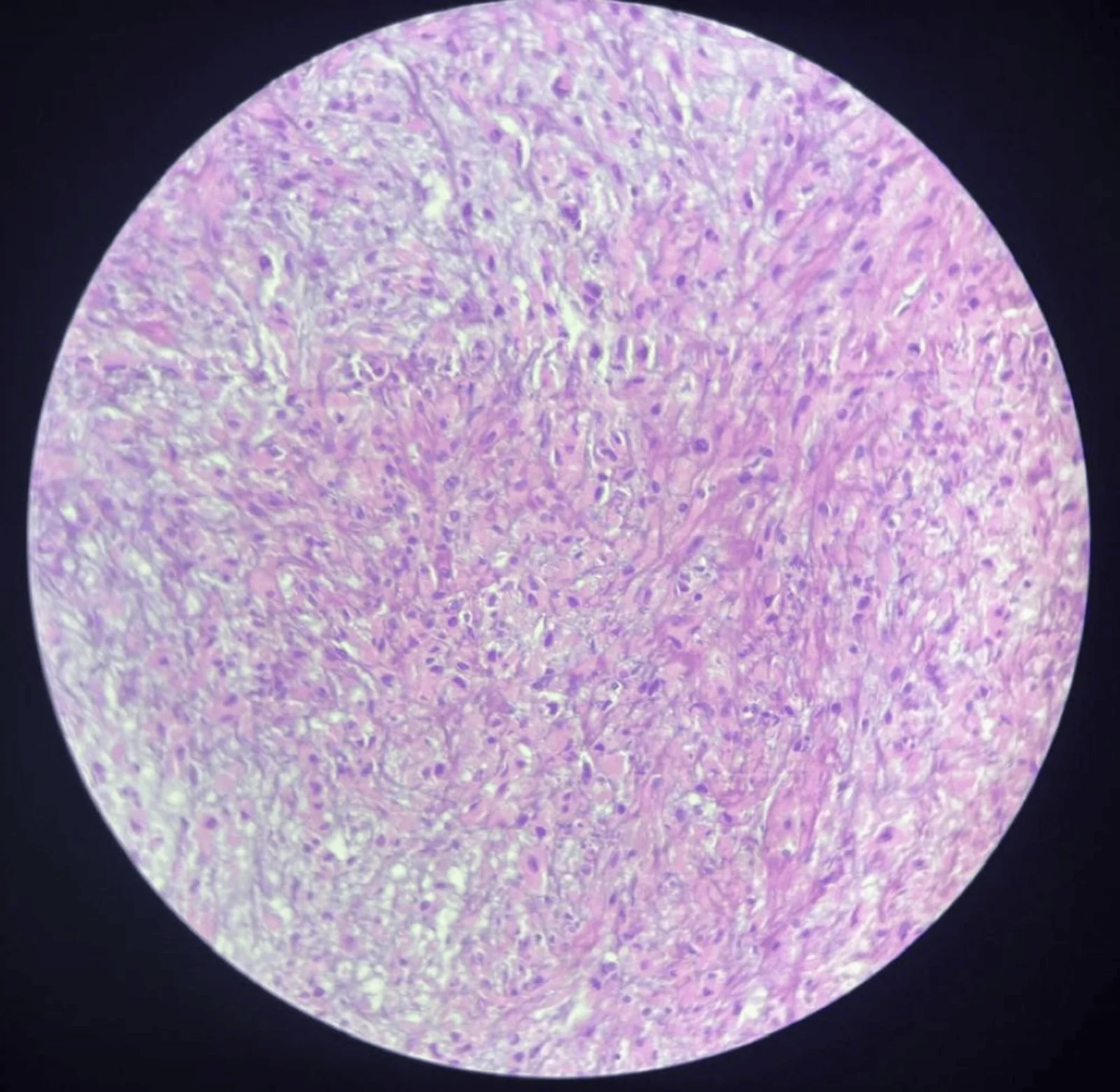
Photomicrograph showing pleomorphic infiltrating glial tumor cells with nuclear atypia in a fibrillary background, consistent with a high-grade diffuse glial neoplasm with glioblastoma-like morphology (H&E, 40×).

**Figure 7:**
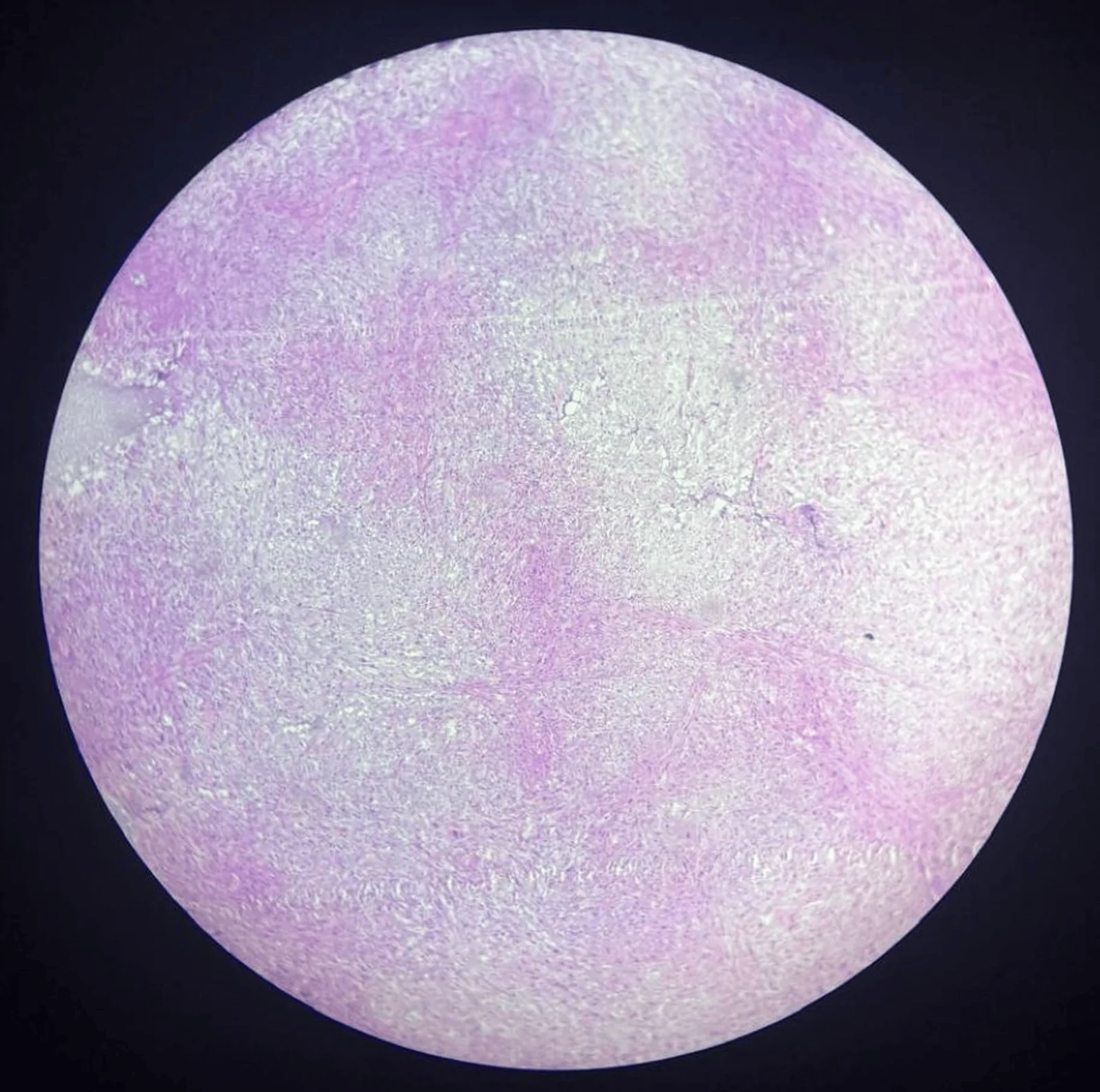
Low-power photomicrograph demonstrating a hypercellular infiltrative glial neoplasm with glioblastoma-like morphology (H&E, 10×).

Grossly, multiple gray-white friable tissue fragments measuring 7 × 4 × 1.5 cm were received. Histopathological examination showed a high-grade malignant neoplasm composed of pleomorphic tumor cells arranged in clusters and scattered singly. The tumor cells demonstrated an increased nuclear-to-cytoplasmic ratio, hyperchromatic nuclei, and occasional epithelioid morphology. Multinucleated giant cells and bizarre tumor forms were present (Figures 6, 7). Necrosis, hemorrhage, and microvascular proliferation were identified. Mitotic activity was approximately 4-5 mitoses per 10 high-power fields.

**Figure 8:**
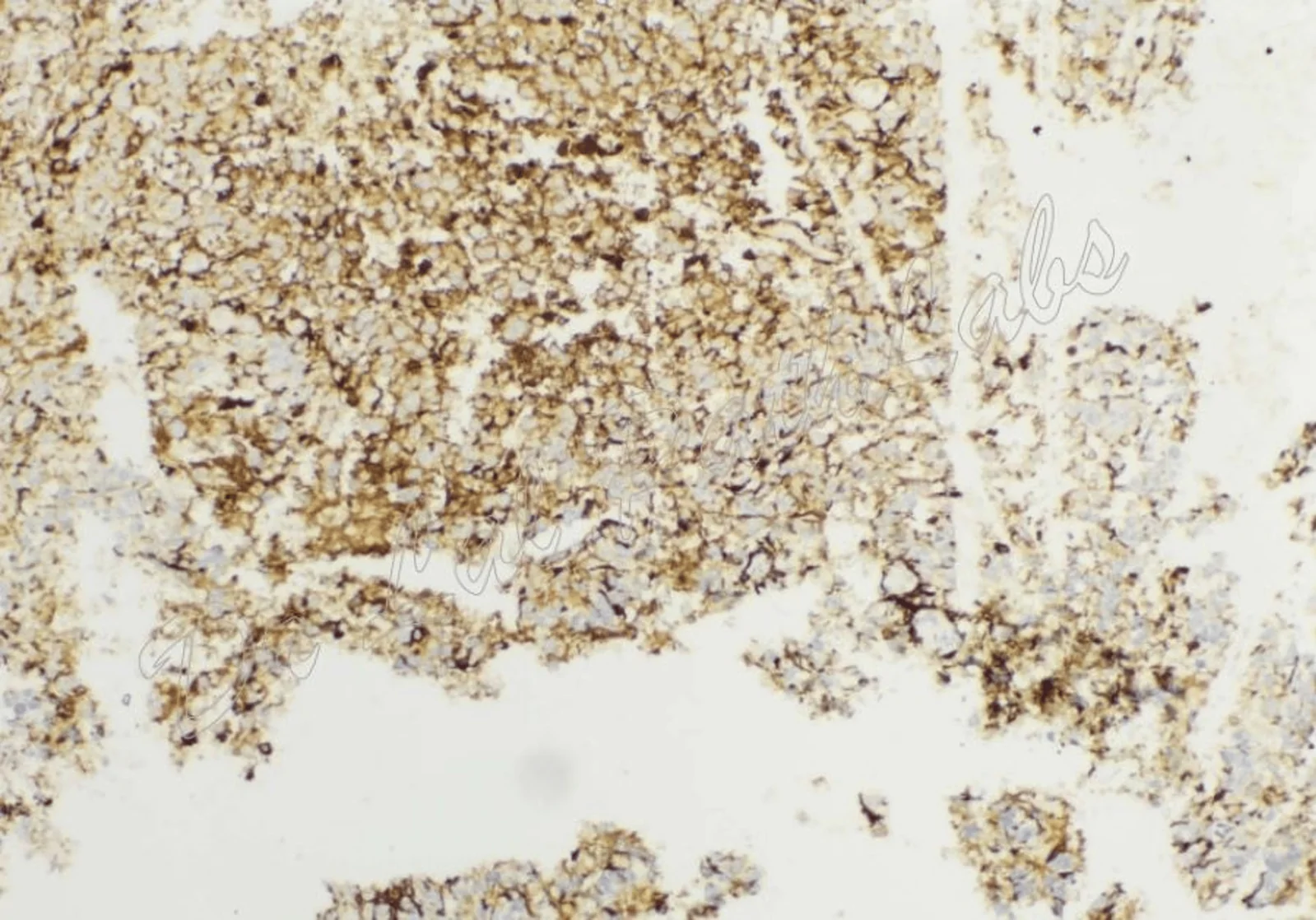
Glial fibrillary acidic protein (GFAP) immunohistochemistry demonstrating diffuse strong cytoplasmic positivity in neoplastic cells, supporting glial differentiation in a high-grade diffuse glial neoplasm with glioblastoma-like morphology (IHC, ×100). The image has been outsourced.

IHC showed positivity for GFAP (Figure 8) and Olig2, supporting glial differentiation.

In the setting of the described histology, the overall features were consistent with a high-grade diffuse glial neoplasm with GBM-like morphology.

IDH1 R132H IHC was not performed in this case. Further integrated classification would depend on IDH status and other molecular parameters, where available.

Case 5

A 60-year-old male presented with headache and progressive neurological symptoms. Neuroimaging revealed an intracranial mass lesion, and a clinical diagnosis of brain tumor was established. He underwent a biopsy of the lesion for histopathological evaluation. Grossly, multiple brownish-white tissue fragments measuring 3 × 2.9 × 1.1 cm were received.

**Figure 9:**
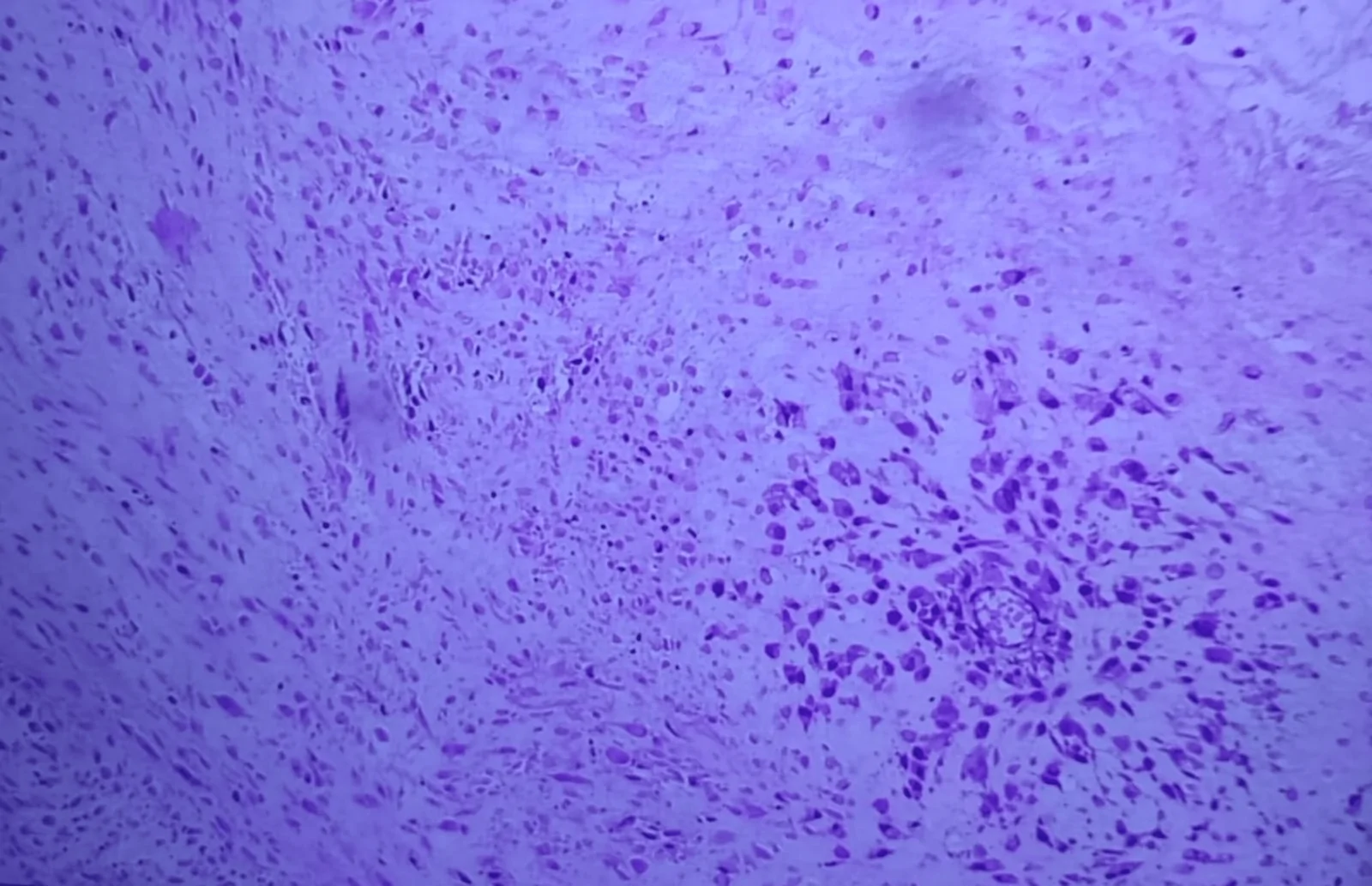
High-power photomicrograph showing a glial neoplasm with perivascular pseudorosettes, characterized by tumor cells radially arranged around blood vessels with intervening fibrillary processes, consistent with ependymoma (H&E, 40×).

Histopathological examination demonstrated a cellular neoplasm composed of round-to-oval tumor cells. Perivascular pseudorosettes (Figure 9) and foci resembling ependymal canals were identified. Areas of necrosis, hemorrhage, and microvascular proliferation were also noted. Occasional mitotic figures were present.

Based on the histomorphological features, the lesion was considered suggestive of a high-grade neuroepithelial tumor, with the differential diagnosis including ependymal tumor and high-grade glial neoplasm with ependymal-like morphology.

An immunohistochemical panel including S-100, GFAP, EMA, D2-40, CD56, and Olig2 was appropriately recommended for further categorization. Final integrated diagnosis should be rendered only after correlation with the complete IHC profile and, where feasible, relevant molecular findings. The clinicopathological, histopathological, immunohistochemical, and integrated WHO CNS5 diagnostic findings of all five cases are summarized in Table 1.

**Table 1:** Summary of clinical, histopathological, immunohistochemical, and integrated WHO CNS5 diagnoses of five adult high-grade glial and related neuroepithelial tumors. Abbreviations: WHO CNS5, World Health Organization Classification of Central Nervous System Tumors, fifth edition; IHC, immunohistochemistry; GFAP, glial fibrillary acidic protein; IDH, isocitrate dehydrogenase; Olig2, oligodendrocyte transcription factor 2; CK, cytokeratin; SOL, space-occupying lesion; ICP, intracranial pressure; GTCS, generalized tonic-clonic seizures.

Case	Age/Sex	Clinical Presentation	Radiology	Histomorphology	IHC Findings	Integrated Diagnosis (WHO CNS5)
1	75/M	Headache, focal deficits, raised ICP	Right frontal SOL	Hypercellular infiltrative neoplasm with pleomorphism, mitoses, pseudopalisading necrosis and hemorrhage	GFAP+, IDH1 R132H+, p53+, Ki-67↑	Astrocytoma, IDH-mutant, CNS WHO grade 4
2	52/M	Seizures, lethargy, headache	Left frontoparietal lesion	Marked pleomorphism, pseudopalisading necrosis, microvascular proliferation, hemorrhage	GFAP+, IDH1 R132H−	Glioblastoma, IDH-wildtype, CNS WHO grade 4
3	84/M	Left frontoparietal SOL	Glioma suspected radiologically	Giant cells, marked pleomorphism, necrosis, microvascular proliferation, brisk mitoses	GFAP+, OLIG2+, IDH1 R132H−, Pan-CK+	High-grade malignant neoplasm with glial differentiation, favoring glioblastoma-like diffuse glial tumor; metastatic carcinoma to be excluded
4	45/M	GTCS, history of neurocysticercosis	Right frontotemporal lesion with satellite focus	Pleomorphic epithelioid tumor cells, giant cells, necrosis, hemorrhage, microvascular proliferation	GFAP+, OLIG2+	High-grade diffuse glial neoplasm with glioblastoma-like morphology
5	60/M	Headache, progressive neurological symptoms	Intracranial mass lesion	Perivascular pseudorosettes, ependymal canal-like structures, necrosis, hemorrhage, microvascular proliferation	GFAP, EMA, D2-40, CD56, OLIG2, S-100 recommended	High-grade neuroepithelial tumor; ependymal tumor versus high-grade glial neoplasm with ependymal-like morphology

## Discussion

The present case series was undertaken to describe the clinicopathological and immunohistochemical spectrum of high-grade glial and related neuroepithelial tumors in adults and to demonstrate the practical application of WHO CNS5 diagnostic principles within the AOSNP-ADAPTR framework in a resource-constrained setting.

All five patients in our series were male, with ages ranging from 45 to 84 years (mean approximately 63 years). Male predominance in high-grade gliomas is well established; Sumathi et al. (2016) [11] reported male preponderance in 61-65% of glioma patients, with hormonal, genetic, and occupational factors postulated as contributing influences. Three of five patients (60%) were in the 40-60 year age group, consistent with the known peak incidence of high-grade diffuse glial tumors in the fifth to seventh decades [1,2]. Presenting symptoms in our cases included headache, seizures, focal neurological deficits, and lethargy, in keeping with the findings of Hamdani et al. (2019) [12], who identified headache and seizures as the most common presenting complaints in glioma patients.

On histopathological evaluation, four of five cases demonstrated features of a diffuse high-grade glial neoplasm, including hypercellularity, marked pleomorphism, necrosis with pseudopalisading, and microvascular proliferation. The fifth case showed perivascular pseudorosettes and ependymal canal-like structures, raising the differential of a high-grade ependymal or glial neoplasm with ependymoid differentiation. GBM-like morphology was the most common impression in our series, which is concordant with published Indian institutional data; Dhar et al. (2014) [13] and Ghanghoria et al. (2014) [14] both reported GBM as the predominant malignant glioma subtype in tertiary care referral populations. Of note, case 4 presented with a background history of neurocysticercosis (NCC). Although a causal link between NCC and glioma has not been established, the co-occurrence in the same patient warrants documentation; the satellite lesion observed radiologically in this case may reflect multifocal GBM rather than residual parasitic disease, and clinicoradiological correlation is essential in such settings.

Immunohistochemical analysis was central to the WHO CNS5-aligned classification in all five cases. In Case 1, IDH1 R132H positivity with concurrent p53 expression and elevated Ki-67 indicated an IDH-mutant diffuse astrocytic neoplasm with grade 4 histological features. Under WHO CNS5, this tumor is appropriately classified as astrocytoma, IDH-mutant, CNS WHO grade 4, and must not be labeled GBM - a critical distinction, as IDH-mutant grade 4 astrocytomas carry a comparatively better prognosis and respond differently to treatment compared to IDH-wildtype GBM [5,6]. ATRX evaluation was recommended for a complete integrated classification in this case. The association between IDH1 mutation and diffusely infiltrating astrocytomas, including its relationship with protein expression and clinical relevance, has been well documented by Thota et al. (2012) [15]. In cases 2 and 4, IDH1 R132H negativity with GFAP and Olig2 positivity was in keeping with GBM, IDH-wildtype, CNS WHO grade 4, consistent with published reports showing IDH-wildtype status in the vast majority of primary GBMs [1,4].

Case 3 presented a notable diagnostic challenge: CK7 positivity was identified alongside GFAP and Olig2 positivity in an elderly male with a high-grade morphology. Aberrant cytokeratin expression has been documented in a small subset of GBMs and must be carefully interpreted in the context of the full immunophenotypic profile and clinical history [3,9]. The combined GFAP and Olig2 positivity in this case supported glial differentiation; however, CK7 positivity mandated exclusion of metastatic epithelial malignancy through expanded IHC (including CK20, EMA, and organ-specific markers) and clinicoradiological correlation. A cautious integrated interpretation - high-grade malignant neoplasm with glial differentiation, metastatic carcinoma excluded - is most appropriate in such settings [10].

Case 5 demonstrated perivascular pseudorosettes and ependymal canal-like structures, raising the differential of a high-grade ependymal neoplasm or a glial tumor with ependymoid differentiation. These morphological features, while suggestive, are not specific to ependymoma and may be encountered in high-grade diffuse glial tumors [7,8]. An IHC panel including GFAP, EMA, D2-40, CD56, Olig2, and S-100 was recommended to confirm ependymal lineage. Necrosis and microvascular proliferation in this case further complicated classification, and a definitive integrated WHO CNS5 diagnosis must await full IHC evaluation and, where feasible, molecular profiling for ZFTA or YAP1 fusions.

The AOSNP-ADAPTR framework proved practically applicable across all five cases through an intermediate resource-level (RL-III/II) diagnostic approach, using GFAP, IDH1 R132H, p53, ATRX, Ki-67, Olig2, EMA, and cytokeratin panels as targeted IHC surrogates. Although advanced molecular testing, such as 1p/19q FISH, TERT promoter analysis, and CDKN2A/B deletion status, was unavailable, the IHC-based approach enabled clinically actionable WHO CNS5-aligned diagnoses. Ebrahimi et al. (2016) [16] demonstrated that IHC-based ATRX assessment reliably predicts IDH and H3F3A mutation status, reinforcing the value of IHC as a surrogate in resource-constrained settings. The Ki-67 proliferation index was elevated in all high-grade diffuse glial cases and served as an additional indicator of tumor aggressiveness, consistent with the observations of Arshad et al. (2010) [17] and Dahlrot et al. (2021) [18], who reported a direct correlation between high Ki-67 expression and tumor grade and worse survival in GBM.

## Conclusions

The present case series demonstrates the clinicopathological and immunohistochemical spectrum of high-grade glial and related neuroepithelial tumors in five adult males evaluated at a tertiary care center. GBM, IDH-wildtype, CNS WHO grade 4 was the predominant impression across the series. Importantly, one case with IDH1 R132H positivity and grade 4 histological features was reclassified as astrocytoma, IDH-mutant, CNS WHO grade 4 under WHO CNS5 criteria, underscoring the critical diagnostic and prognostic significance of IDH1 IHC. A morphologically challenging case with aberrant CK7 expression required expanded IHC to exclude metastatic carcinoma before glial differentiation could be confirmed. The case with perivascular pseudorosettes and ependymal canal-like structures illustrated the need for a dedicated IHC panel before a definitive integrated diagnosis can be assigned to tumors with ependymoid morphology. The AOSNP-ADAPTR framework provides a practical, structured, and reproducible approach for implementing the WHO CNS5 classification using targeted IHC panels in resource-constrained settings, enabling clinically meaningful tumor characterization without comprehensive molecular profiling.
